# Pre-Ophthalmoscopic Quantitative Biomarkers in Diabetes Mellitus

**DOI:** 10.1167/tvst.12.3.24

**Published:** 2023-03-27

**Authors:** Zsofia Kolkedi, Adrienne Csutak, Eszter Szalai

**Affiliations:** 1 Department of Ophthalmology, University of Pécs Medical School, Pécs, Hungary

**Keywords:** optical coherence tomography (OCT) angiography, confocal microscopy, neuropathy, diabetes mellitus (DM)

## Abstract

**Purpose:**

The purpose of this study was to assess whether retinal microvascular or corneal nerve abnormalities occur earlier in diabetes mellitus (DM) and to identify imaging biomarkers in order to help prevent the subsequent irreversible retinal and corneal complications.

**Methods:**

The study comprised 35 eyes of 35 healthy volunteers and 52 eyes of 52 patients with type 1 and type 2 DM. Swept-source optical coherence tomography (OCT), OCT angiography, and in vivo corneal confocal microscopy were performed in both groups. Corneal sub-basal nerve plexus and vessel density (VD) of superficial capillary plexus (SCP) and deep capillary plexus (DCP) were evaluated.

**Results:**

All corneal sub-basal nerve fiber parameters were decreased in patients with DM compared with healthy subjects and the difference was significant for each result except for nerve fiber width (*P* = 0.586). No significant correlation was obtained between any nerve fiber morphology parameters and disease duration or HbA1C. VD in SCP was significantly decreased in the superior (*P* < 0.0001), temporal (*P* = 0.001), and nasal quadrant (*P* = 0.003) in the diabetes group. In DCP, only superior VD (*P* = 0.036), decreased significantly in the diabetes group. Ganglion cell layer thickness in the inner ring showed a significantly lower value in patients with DM (*P* < 0.0001).

**Conclusions:**

Our results implicate a more pronounced and earlier damage to the corneal nerve fibers compared to the retinal microvasculature in patients with DM.

**Translational Relevance:**

In DM, an earlier and more pronounced damage to the corneal nerve fibers was observed compared to the retinal microvasculature.

## Introduction

Small fiber polyneuropathy and retinopathy are long-term microvascular complications of diabetes mellitus (DM). The worldwide diabetic retinopathy (DR) population has been estimated to increase by 55.6% from 2020 to 2045.^1^ This can be attributed to the rapidly expanding diabetic population. The UK Prospective Diabetes Study (UKPDS) presented that up to 40% of patients with type 2 diabetics (T2DM) have some degree of DR at the time of diagnosis; however, in case of type 1 diabetes (T1DM), DR is considered as a later and more acute complication.^2^ Peripheral neuropathy can be detected both in T1DM, T2DM, as well as in prediabetic states (impaired glucose tolerance and impaired fasting glucose) and metabolic syndrome.^3^^,^^4^ The UKPDS investigation and more recent population-based studies have shown that strict glucose control and improved cardiovascular status may reduce the risk of the DM-related tissue complications, including retinopathy and neuropathy, and allow for prevention of further morbidity and mortality.^5^^,^^6^

Regular ophthalmic screening is recommended for patients with DM to monitor DR and other ocular complications. However, imaging technologies have been evolving rapidly that may assist or possibly replace the standard ophthalmoscopic examination. Recently, ophthalmologists are able to qualify and quantify the retinal capillary network and choriocapillaries, optic nerve head, and corneal peripheral nerves. In vivo confocal microscopy (IVCM) has been shown to reveal early corneal sub-basal nerve fiber changes in patients with diabetes before any apparent funduscopic alterations.^7^^,^^8^ Optical coherence tomography angiography (OCTA) provides a direct, noninvasive visualization and quantification of the retinal microvasculature and blood flow.^9^

A biomarker is a feature that can be evaluated objectively and is of importance in diagnosis, grading, and prognosis of a particular disease.^10^ A quantitative imaging biomarker can be defined as an objective indicator of a pathogenic process.^11^ Our aim was to assess whether retinal microvascular or corneal nerve abnormalities occur earlier in DM and to identify imaging biomarkers in order to help prevent the subsequent irreversible retinal and corneal complications.

### Patients and Methods

This cross-sectional study comprised 35 eyes of 35 healthy volunteers and 52 eyes of 52 patients with T1DM (10 patients) and T2DM (42 patients). A complete ophthalmic examination was carried out on every study subject, including visual acuity, intraocular pressure, slit-lamp examination with dilated fundus examination, corneal tomography (Anterion; Heidelberg Engineering GmbH, Heidelberg, Germany), IVCM (Heidelberg Retina Tomograph II Rostock Cornea Module; Heidelberg Engineering GmbH, Heidelberg, Germany), posterior segment optical coherence tomography (OCT) and OCTA (Topcon DRI OCT Triton Swept source OCT, Topcon, Japan). Healthy subjects had a negative history of ocular surgery, trauma, and present or prior ophthalmic disease other than refractive errors (less than ± 3.0 D spherical and cylindrical power). Dilated fundus investigation was performed in all study patients. The International Clinical Diabetic Retinopathy Disease Severity Scale^12^ was used to classify the stage of DR: 0 = no retinopathy, 1 = mild nonproliferative DR, 2 = moderate nonproliferative DR, 3 = severe nonproliferative DR, and 4 = proliferative DR. The study was performed in accordance with the tenets of the Helsinki Declaration and the protocol was approved by the University of Pecs Institutional Ethical Review Board (Number: 8433 – PTE 2020.). Written informed consent was obtained from all study subjects.

All study subjects underwent IVCM of all corneal layers, as described previously.^8^^,^^13^ In both study groups, one eye was selected randomly for further analysis. Three good quality images of the sub-basal nerve plexus were selected in three different areas of the central cornea and they were analyzed with ACCMetrics software version 3 (University of Manchester, Manchester, UK).^14^^–^^18^ Corneal nerve fiber density (NFD), the number of nerve fibers/mm^2^; nerve branch density (NBD), the number of primary branch points on the main nerve fibers/mm^2^; nerve fiber length (NFL), the total length of nerves mm/mm^2^; nerve fiber total branch density (TBD), the total number of branch points/mm^2^, nerve fiber area (NFA), the total nerve fiber area mm^2^/mm^2^; and nerve fiber width (NFW), the average nerve fiber width mm/mm^2^, and fractal dimension (FD) were evaluated.

OCTA imaging was performed with 3 mm × 3 mm volumetric scans centered at the fovea. Automated layer segmentation was obtained for superficial capillary plexus (SCP) and deep capillary plexus (DCP) using the instrument-based software (IMAGEnet 6 version 1.26.16898; Topcon), as described previously.^19^ The vessel density (VD) was measured in four quadrants by IMAGEnet software. Foveal avascular zone (FAZ) was manually outlined by the same trained examiner (author Z.K.) using the built-in software. Structural OCT was performed with SMARTTrack HD Raster centered at the macula (6.0 × 6.0 mm) and 3D Disc program (6.0 × 6.0 mm) centered at the optic nerve head. Retinal nerve fiber layer thickness (RNFL) between inner limiting membrane (ILM)-RNFL/ganglion cell layer (GCL) boundaries centered on the optic disc and central choroidal thickness between Bruch's membrane and choroid-sclera interface centered at the macula were evaluated by using the instrument-generated thickness maps. For ganglion cell complex thickness measurement, two values were recorded centered at the macula: GCL+ between RNFL/GCL-inner plexiform layer (IPL)/inner nuclear layer (INL) boundaries and GCL++ between ILM-IPL/INL boundaries. Central retinal thickness was also measured between the ILM-outer segment/retinal pigment epithelium boundaries in the macula. All IVCM and OCT examinations were acquired by an experienced examiner (ZK). The image selection and analysis for the IVCM and OCTA were carefully reviewed by two independent examiners (authors Z.K. and E.S.). Low-quality IVCM and OCTA images or presence of any motion artifacts were excluded from the analysis.

### Statistical Analysis

Data were analyzed using the SPSS Statistics 25.0 (IBM Corp., Armonk, NY), MedCalc version 14.8.1 (MedCalc Software, Ostend, Belgium), and Prism 9.4.1 for macOS (GraphPad Software, San Diego, CA, USA). For each data set, mean, standard deviation (SD) and 95% confidence interval (95% CI) for the mean were calculated. Mann-Whitney *U* test was carried out for comparison between the two groups or variables. For bivariate correlation analysis, the Spearman's rank correlation “r” was used. A *P* value below 0.05 was considered statistically significant.

## Results

The mean age of the healthy volunteers and patients with DM was 49.88 ± 17.01 years (ranging from 20 to 73 years) and 56.04 ± 13.66 years (ranging from 22 to 78 years), respectively (*P* = 0.155). In the diabetes group, the mean disease duration was 11.17 ± 11.73 years (ranging from 1 month to 52 years). The mean HbA1c was 7.28% ± 1.33% (ranging from 5.5% to 13%). The mean body mass index for patients with DM was 31.01 ± 6.28 (ranging from 21.26 to 47.45). T1DM was diagnosed in 10 patients and T2DM was found in 42 patients. No subjects had moderate to severe nonproliferative or proliferative DR.

The ocular biometry data of all subjects are summarized in Table 1. No statistically significant difference was found in any of the biometry results between the two groups except for the central corneal thickness (*P* = 0.016) and lens thickness (*P* = 0.018).

**Table 1.** Ocular Biometry in Healthy Volunteers Compared to Patients With Diabetes Mellitus

	Healthy Volunteers^a^	Patients With Diabetes Mellitus^a^	*P* Value^b^
Anterior K1 (D)	43.436 ± 1.593	43.258 ± 1.391	0.341
	(42.872 − 44.001)	(42.773 − 43.743)	
Anterior K2 (D)	44.157 ± 1.720	44.011 ± 1.469	0.319
	(43.547 − 44.767)	(43.499 − 44.524)	
Astigmatism (D)	0.912 ± 0.628	0.735 ± 0.372	0.454
	(0.693 − 1.131)	(0.603 − 0.867)	
CCT (µm)	545.625 ± 50.365	563.618 ± 42.546	0.016
	(527.466 − 563.784)	(548.773 − 578.462)	
Internal ACD (mm)	2.757 ± 0.555	2.83 ± 0.564	0.267
	(2.560 − 2.953)	(2.627 − 3.034)	
WTW (mm)	11.858 ± 0.406	11.763 ± 0.395	0.379
	(11.712 − 12.004)	(11.625 − 11.901)	
LT (mm)	4.262 ± 0.363	4.506 ± 0.388	0.018
	(4.134 − 4.391)	(4.355 − 4.656)	
AL (mm)	23.373 ± 1.113	23.197 ± 0.918	0.677
	(22.978 − 23.767)	(22.871 − 23.522)	

K, keratometry; CCT, central corneal thickness; ACD, anterior chamber depth; WTW, white-to-white; LT, lens thickness; AL, axial length.

a Mean ± standard deviation (95% confidence interval).

b Mann-Whitney *U* test.

**Figure 1.**
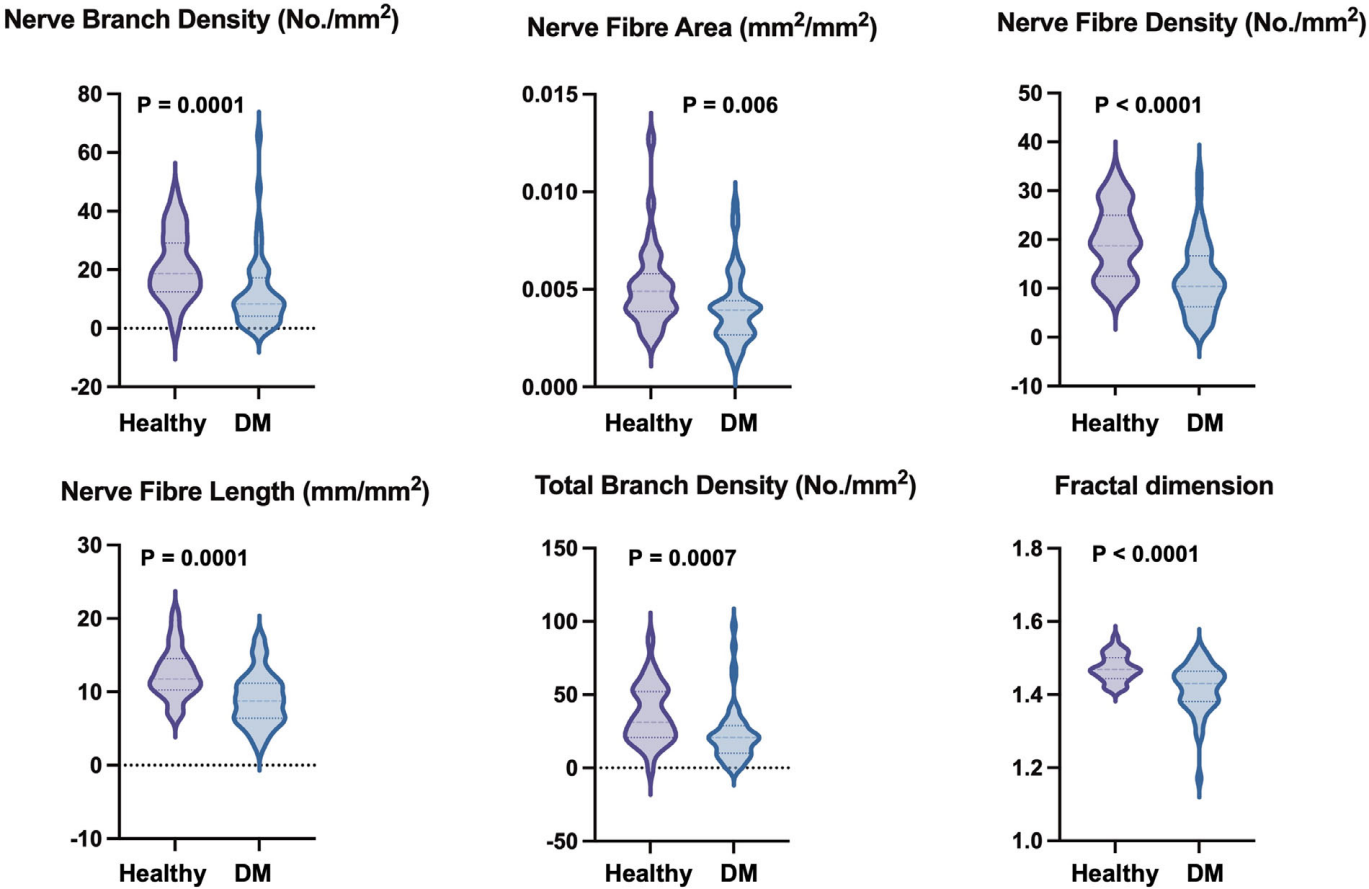
Violin plots of the median and quartiles showing frequency distribution of the sub-basal nerve fiber morphology data in healthy subjects and patients with diabetes mellitus (DM).

**Figure 2.**
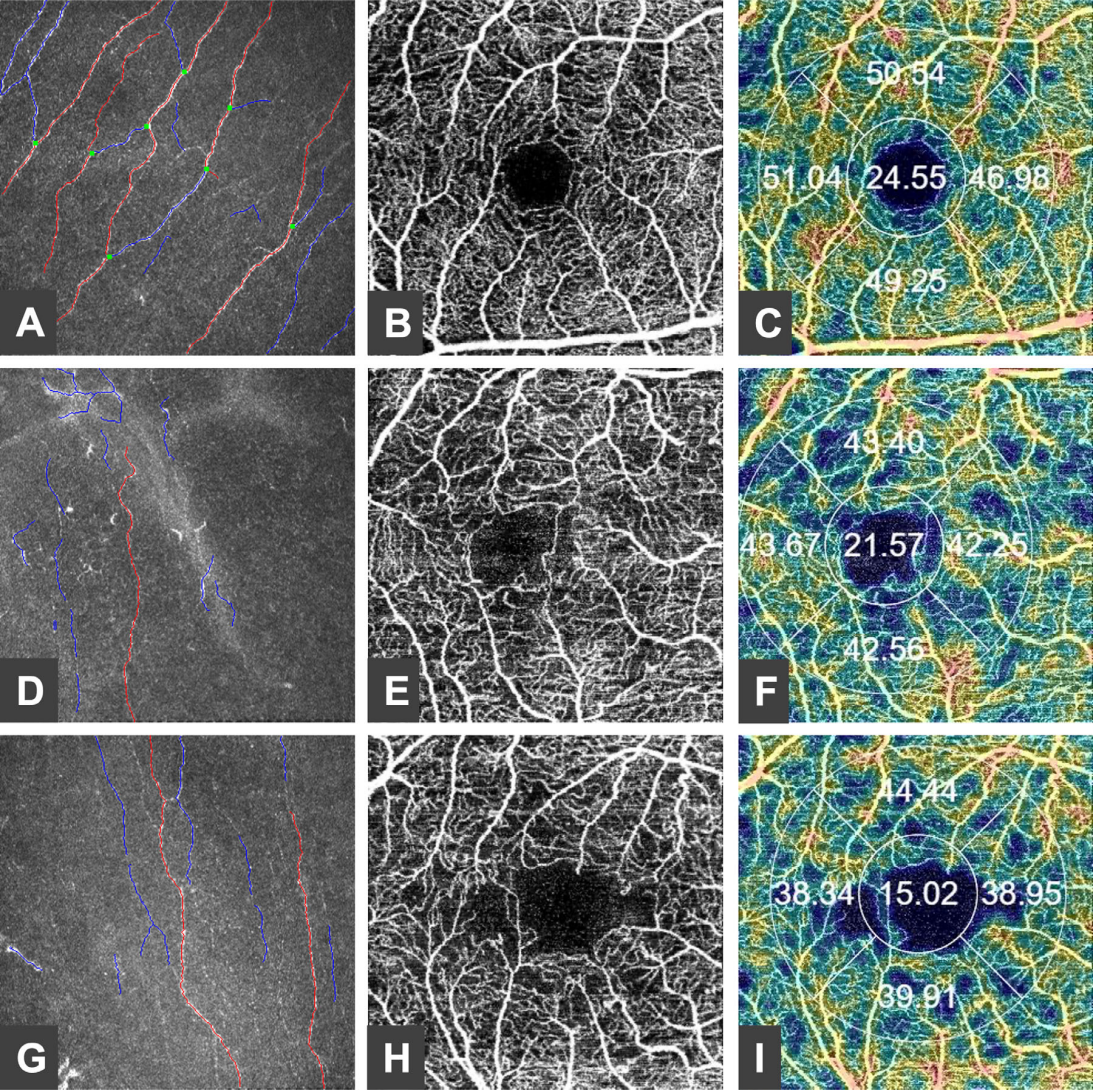
Annotated image of the subbasal nerve plexus using in vivo confocal microscopy (*red*, nerve fiber; *blue*, nerve branch; and *green*, nerve branch point) (**A, D, G**). Optical coherence tomography angiography (OCTA) centered at the fovea showing the foveal avascular zone (FAZ) (**B, E, H**). Vessel density (VD) map in the superficial capillary plexus in four quadrants (**C, F, I**). Normal nerve fiber morphology (**A**), OCTA (**B**), and VD map (**C**) of a healthy volunteer. Altered nerve fiber morphology (**D**), impaired FAZ circularity (**E**), and decreased VD (**F**) of a 44-year-old patient with type 1 diabetes mellitus for 40 years without retinopathy (HbA1c, 7.67%). Sub-basal nerve fiber damage (**G**), enlarged FAZ (**H**), and decreased VD (**I**) in a 49-year-old patient with type 2 diabetes mellitus for 10 years without retinopathy (HbA1c, 9.11%).

All corneal subbasal nerve fiber parameters were decreased in patients with DM compared to healthy subjects (Figs. 1, 2, Table 2) and the difference was significant for each result except for NFW (*P* = 0.586). No statistically significant correlation was obtained between any nerve fiber morphology parameters and disease duration or HbA1C.

**Table 2.** Corneal Nerve Fiber Morphology of Healthy Volunteers Compared to Patients With Diabetes Mellitus

	Healthy Subjects^a^	Patients With Diabetes Mellitus^a^	*P* Value^b^
Nerve branch density (No/mm^2^)	21.252 ± 11.414	12.095 ± 12.663	0.0001
	(17.065 − 25.439)	(8.496 − 15.694)	
Nerve fiber area (mm^2^/mm^2^)	0.005 ± 0.002	0.004 ± 0.002	0.006
	(0.004 − 0.006)	(0.004 − 0.005)	
Nerve fiber density (No/mm^2^)	19.723 ± 7.216	12.016 ± 7.192	<0.0001
	(17.076 − 22.370)	(9.972 − 14.060)	
Nerve fiber length (mm/mm^2^)	12.489 ± 3.389	9.041 ± 3.584	0.0001
	(11.245 − 13.732)	(8.022 − 10.059)	
Nerve fiber width (mm/mm^2^)	0.022 ± 0.002	0.021 ± 0.002	0.586
	(0.021 − 0.022)	(0.021 − 0.022)	
Nerve fiber total branch density (No/mm^2^)	36.49 ± 19.740	22.965 ± 19.625	0.0007
	(29.249 − 43.730)	(17.388 − 28.542)	
Nerve fiber fractal dimension	1.473 ± 0.036	1.418 ± 0.066	<0.0001
	(1.460 − 1.486)	(1.399 − 1.437)	

a Mean ± standard deviation (95% confidence interval).

b Mann-Whitney *U* test.

**Figure 3.**
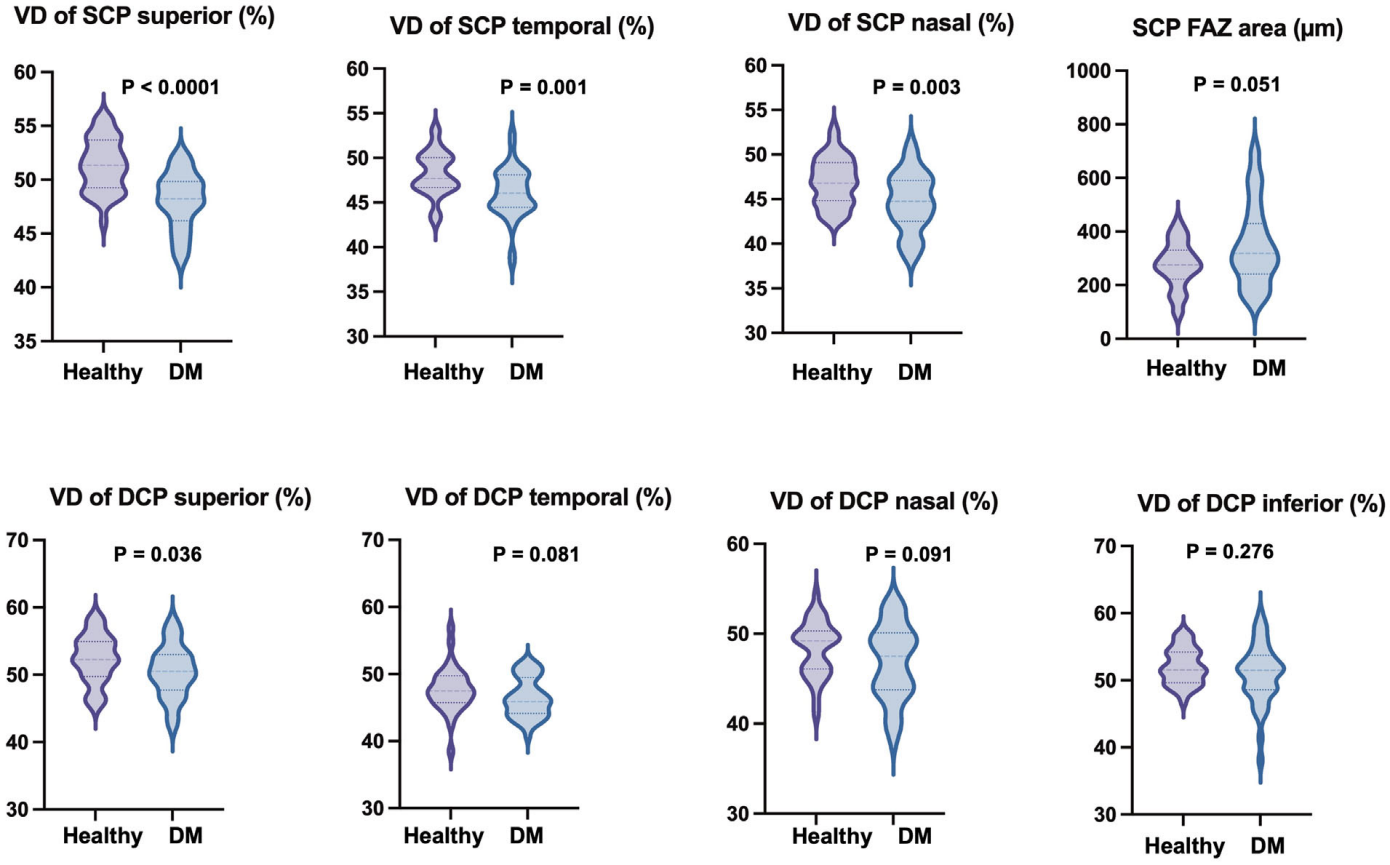
Violin plots of the median and quartiles showing frequency distribution of the vessel density (VD) data in the superficial (SCP) and deep capillary plexus (DCP) and foveal avascular zone (FAZ) area in healthy subjects and patients with diabetes mellitus (DM).

No significant difference was observed in central retinal thickness between the control and diabetes groups (*P* = 0.089; Table 3). Central choroidal thickness was lower in patients with DM (*P* = 0.016). The vessel density in SCP was significantly decreased in the superior (*P* < 0.0001), temporal (*P* = 0.001), and nasal quadrant (*P* = 0.003) in the diabetes group (Figs. 2, 3). In DCP, only superior VD (*P* = 0.036), decreased significantly in the diabetes group (see Fig. 3). GCL in the inner ring showed a significantly lower value in patients with DM (*P* < 0.0001). There was an enlargement in the FAZ area measured in SCP in the diabetes group but the difference was only borderline compared to the healthy group (*P* = 0.051). Significant inverse correlation was observed between disease duration and vessel density of SCP in superior (r = −0.539, *P* < 0.0001), temporal (r = −0.557, *P* < 0.0001), inferior (r = −0.433, *P* = 0.005), and nasal quadrant (r = −0.372, *P* = 0.015) and between disease duration and vessel density of DCP in the inferior (r = −0.369, *P* = 0.019) and nasal quadrants (r = −0.458, *P* = 0.003). HbA1C did not show significant correlation with any of the OCTA parameters. No significant correlation was observed between any of the sub-basal nerve fiber morphology and retinal microvasculature parameters in the study groups.

**Table 3.** Optical Coherence Tomography (OCT) and OCT Angiography Parameters in Healthy Volunteers Compared to Patients With Diabetes Mellitus

	Healthy Subjects^a^	Patients With Diabetes Mellitus^a^	*P* Value^b^
Central retinal thickness (µm)	259.029 ± 18.978	251.0 ± 24.491	0.089
	(252.408 − 265.651)	(244.182 − 257.818)	
Central choroidal thickness (µm)	273.455 ± 72.974	237.708 ± 81.935	0.016
	(247.579 − 299.330)	(213.917 − 261.500)	
VD of SCP CSF (%)	21.649 ± 5.442	19.88 ± 5.219	0.116
	(19.780 − 23.519)	(18.274 − 21.486)	
VD of SCP superior (%)	51.377 ± 2.449	48.063 ± 2.645	<0.0001
	(50.535 − 52.218)	(47.239 − 48.887)	
VD of SCP temporal (%)	48.207 ± 2.456	46.069 ± 2.786	0.001
	(47.364 − 49.051)	(45.212 − 46.927)	
VD of SCP inferior (%)	50.990 ± 3.067	49.147 ± 3.910	0.138
	(49.920 − 52.061)	(47.929 − 50.366)	
VD of SCP nasal (%)	46.937 ± 2.689	44.691 ± 3.307	0.003
	(46.013 − 47.861)	(43.674 − 45.709)	
SCP FAZ area (µm)	274.722 ± 86.541	348.511 ± 151.528	0.051
	(244.036 − 305.408)	(301.292 − 395.731)	
VD of DCP CSF (%)	19.317 ± 4.632	17.843 ± 5.460	0.236
	(17.647 − 20.988)	(16.142 − 19.545)	
VD of DCP superior (%)	52.359 ± 3.596	50.483 ± 3.890	0.036
	(51.124 − 53.594)	(49.255 − 51.710)	
VD of DCP temporal (%)	47.673 ± 3.302	46.485 ± 2.943	0.081
	(46.539 − 48.808)	(45.568 − 47.402)	
VD of DCP inferior (%)	52.072 ± 2.684	50.924 ± 4.420	0.276
	(51.150 − 52.994)	(49.529 − 52.319)	
VD of DCP nasal (%)	48.345 ± 3.054	46.855 ± 4.166	0.091
	(47.296 − 49.394)	(45.557 − 48.154)	
GCL ++ CSF (µm)	60.379 ± 11.723	64.901 ± 22.679	0.598
	(56.291 − 64.472)	(58.310 − 71.475)	
GCL ++ inner ring (µm)	116.904 ± 7.699	109.224 ± 9.628	<0.0001
	(114.218 − 119.591)	(106.428 − 112.020)	
GCL ++ outer ring (µm)	108.794 ± 6.920	104.972 ± 10.129	0.037
	(106.380 − 111.208)	(102.031 − 107.913)	
GCL+ CSF (µm)	53.235 ± 9.433	55.042 ± 15.612	0.595
	(49.944 − 56.526)	(50.509 − 59.575)	
GCL + inner ring (µm)	90.941 ± 6.326	83.76 ± 8.009	<0.0001
	(88.734 − 93.148)	(81.435 − 86.086)	
GCL + outer ring (µm)	67.36 ± 6.803	64.86 ± 5.894	0.152
	(64.987 − 69.734)	(63.129 − 66.590)	
RNFL total thickness (µm)	106.667 ± 12.504	101.848 ± 10.556	0.060
	(102.233 − 111.100)	(98.713 − 104.982)	

VD, vessel density; SCP, superficial capillary plexus; DCP, deep capillary plexus; FAZ, foveal avascular zone; GCL, ganglion cell layer; RNFL, retinal nerve fiber layer; CSF, thickness within central 1 mm, inner ring: thickness within central 3 mm, outer ring: thickness within central 6 mm.

a Mean ± standard deviation (95% confidence interval).

b Mann-Whitney *U* test.

## Discussion

In this study, we examined patients with diabetes with no to mild ophthalmoscopic alterations using IVCM and OCTA to assess whether retinal microvascular or corneal nerve abnormalities occur earlier in DM. Altered sub-basal nerve fiber morphology was found in patients with DM even in the lack of DR. Previous studies found NFL as the best parameter for diagnosing diabetic peripheral neuropathy followed by NFD.^7^^,^^20^ Brines et al. considered NFL and NFA to be more sensitive indicators of mild neuropathy.^21^ Fractal dimension is a relatively new parameter to evaluate corneal nerve morphology by quantifying the spatial loss of nerve fibers. FD was also proposed to have the ability to differentiate between distinct neuropathy etiologies.^22^ A multinational study suggested that IVCM had a potent predictive diagnostic ability to identify patients with diabetes with greater risk for further diabetic polyneuropathy.^23^ In our previous study in young patients with T1DM, we observed lower TBD, confirming early more distal loss of nerve branches, consistent with this loss of thinner more distal branches. Total NFA was comparable between control subjects and patients with diabetes, presumably due to the early relative preservation of main nerve fibers with a primary reduction in nerve branches.^8^ In the present study, NFW did not differ significantly between diabetic and healthy subjects supporting our previous explanation on early more pronounced loss of distal nerve branches.

In our study, retinal vessel density in SCP was significantly decreased in the superior, temporal, and nasal quadrant in patients with diabetes. In DCP, only superior vascular density decreased significantly in patients with DM. Ong et al. observed lower VD in SCP with higher DR severity in the no retinopathy to mild nonproliferative DR groups and concluded that SCP VD changes may be more sensitive for discriminating nonproliferative DR.^24^ Previous authors also demonstrated that in patients with a long history of T2DM without DR, a decrease in VD and perfusion and impairment of the FAZ border occur earlier in the superficial vascular plexus than in the deep vascular plexus.^25^ In contrast, Kaoual et al. described retinal vascular alterations in patients with no to early retinopathy primarily in DCP suggesting that the deeper vascular plexus is more vulnerable to DM severity vascular changes.^26^ In contrast to our findings, Kirthi et al. observed more prominent retinal microvascular changes in prediabetes compared to corneal nerve fiber damage, although the central corneal NFL showed significantly decreased values in T2DM when compared to normoglycemic subjects.^27^ They highlight the impact of obesity, waist size, and body mass index (BMI) on corneal nerve loss.^27^

According to other authors, we detected early retinal microvascular changes with OCTA in the absence of ophthalmoscopic alterations.^28^^,^^29^ Ghassemi et al. identified parafoveal VD of SCP as a predicting biomarker for visual impairment in DM.^30^ Significant FAZ increase in SCP was also observed in our patients with diabetes. The ganglion cell complex thickness was significantly decreased in DM in our study. Accordingly, Qiu et al. described correlations between ganglion cell body loss and retinal vasculature changes with the severity of DR.^31^ Previous papers highlighted the association between neuronal degeneration and microvascular changes in diabetes mellitus.^31^^,^^32^ GCL and RNFL thickness changes in early DM have been identified as neuroretinal degeneration that may anticipate microvascular alterations.^32^ In a recent study, a clear interaction was observed between age or age at diagnosis and diabetes duration and the risk of microvascular events. They found the greatest risks of microvascular events in the youngest ages with the longest disease duration.^33^

There are a few limitations to this study. First, this was a simultaneous measurement of exposure (DM) and outcome, it is difficult to derive causal or temporal relationships from a cross-sectional analysis and it might not be representative of the diabetic population. Second, there was a small sample size of patients with DR, a larger case number with more advanced ophthalmoscopic changes would be required. Third, the ophthalmic imaging was performed at a time point during the disease course, not at baseline, and in patients with diabetes it is difficult to know the exact disease duration. Fourth, snapshots of the sub-basal corneal nerves were selected manually, they only represent a small part of the cornea that might have some subjective elements.

In summary, sub-basal nerve fiber morphology was altered significantly in patients with DM but they did not show a significant correlation with disease duration and HbA1c. Our results implicate a more pronounced and earlier damage to the corneal nerve fibers compared to the retinal microvasculature in patients with DM.
